# How to Cleanse the Waste Pipes

**Published:** 1883-12

**Authors:** 


### HOW TO CLEANSE THE WASTE PIPES.

ONE of the most frequent and trying annoyances of housekeeping, as many can testify, and which a writer in the Philadelphia *Ledger* freely asserts, is the obstruction to the free, quick outlet of the waste water of the washstand, the bathtub and the kitchen sink.

This is caused by a gradual accumulation of small bits of refuse material, paper, rags, meat, bones, or other offal, which check and finally entirely stop the outflow of the waste water; and then the plumber is called to remove the stoppage with his force-pump.

Sometimes this is effective, at others the offending waste pipe is cut out and a new one put in its place at considerable cost.

But the plumber is not always near at hand or free to come at one's call, and the matter demands immediate attention. A simple, inexpensive method of clearing the pipe is as follows: Just before retiring at night pour into the pipe enough *liquid* potash lye of 36 degrees strength to fill the "trap," as it is called, or bent portion of the pipe just below the outlet. About a pint will suffice for a washstand, or a quart for a bathtub or kitchen sink. *Be sure that no water runs into it till next morning.*

During the night the lye will convert all of the offal in the pipe into *soft soap*, and the first current of water in the morning will remove it entirely and leave the pipe as clean as new. The writer has never had occasion, in over thirty years' experience, to make more than two applications of it in any one case.

A remarkable example of the value of this process was that of a large drain pipe which carried off the waste of an extensive country house, near Philadelphia, and ran under a beautiful lawn in its front. A gallon of the lye removed all obstruction in a single night, and saved the necessity of digging up the pipe and disfiguring the greensward of the lawn, as the plumber intended, until advised of this process.

The so-called potash lye sold in small tin cans in the shops is not recommended for this purpose; it is quite commonly misnamed, and is called caustic *soda*, which makes a *hard* soap. The lye should be kept in heavy glass bottles or demijohns, covered with wicker work, and plainly labeled; always under lock when not in actual use. It does not act upon metals, and so does not corrode the pipes as do strong acids.

---

To quiet the burning of ivy-poisoned hands, wet them with hot lime-water. This will be efficacious sometimes when nothing else does any good.

